# Functional Evolution of a *cis-*Regulatory Module

**DOI:** 10.1371/journal.pbio.0030093

**Published:** 2005-03-15

**Authors:** Michael Z Ludwig, Arnar Palsson, Elena Alekseeva, Casey M Bergman, Janaki Nathan, Martin Kreitman

**Affiliations:** **1**Department of Ecology and Evolution, University of ChicagoIllinoisUnited States of America; **2**Department of GeneticsUniversity of CambridgeUnited Kingdom; University of California, BerkeleyUnited States of America

## Abstract

Lack of knowledge about how regulatory regions evolve in relation to their structure–function may limit the utility of comparative sequence analysis in deciphering *cis-*regulatory sequences. To address this we applied reverse genetics to carry out a functional genetic complementation analysis of a eukaryotic *cis-*regulatory module—the *even-skipped* stripe 2 enhancer—from four *Drosophila* species. The evolution of this enhancer is non-clock-like, with important functional differences between closely related species and functional convergence between distantly related species. Functional divergence is attributable to differences in activation levels rather than spatiotemporal control of gene expression. Our findings have implications for understanding enhancer structure–function, mechanisms of speciation and computational identification of regulatory modules.

## Abstract 2

Analysis of the *even-skipped* stripe 2 enhancer from four *Drosophila* species revealed that functional divergence is attributable to differences in activation levels rather than spatiotemporal control of gene expression

## Introduction

The annotation of genes from comparative sequence data rests on a fundamental evolutionary dictum, first elaborated by M. Kimura, that the rate of molecular evolution will be inversely related to the level of functional constraint. But the application of this principle would not be interpretable without a corresponding understanding of gene structure and organization (i.e., the genetic code and its degeneracy, the signals for initiation and termination of translation, intron/exon junction sequences, etc.). Knowledge of equivalent scope and depth does not exist for *cis-*regulatory sequences. These sequences often contain docking sites for transcription factors (TFs), but the number of binding sites and the spacing between them vary, and binding-site sequences are often degenerate to the point that they can only be characterized probabilistically. Even more striking is the lack of data relating functional evolution of gene expression to *cis-*regulatory sequence evolution. There are good reasons to expect the two may be only weakly correlated [1,2]: De novo binding sites can readily evolve [3]; individual TFs often bind at multiple locations and may be exchangeable, and the spacing between binding sites can rapidly evolve. Thus, despite recent progress [4,5], rules have yet to be elucidated for the functional molecular evolution of this critically important component of the genome.

**Figure 1:**
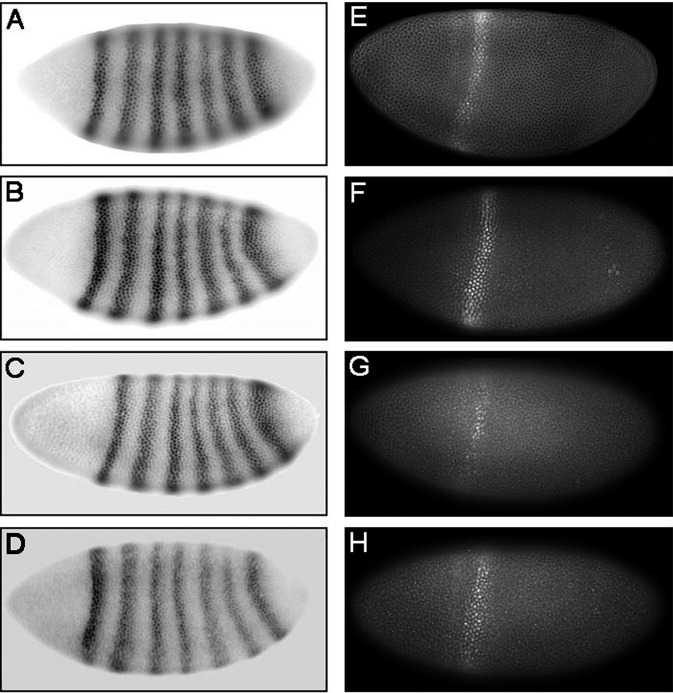
Expression of *eve* (A–D) Embryos of four *Drosophila* species at early cellular blastoderm stage. EVE stained with immunoperoxidase DAB reaction enhanced by nickel. (E–H) *Df(eve) D. melanogaster* embryos with two copies of transgenes containing *eve* S2E from four species fused to *D. melanogaster eve* coding region (−0.9 to +1.85 kb) at blastoderm stage. Immunofluorescence-labeled EVE. The *S2E_ere_-EVE* (G) produces consistently weaker stripes than lines carrying S2Es from the other three species. (A and E) *D. melanogaster,* (B and F) *D. yakuba,* (C and G) *D. erecta,* and (D and H) *D. pseudoobscura.*

**Figure 2:**
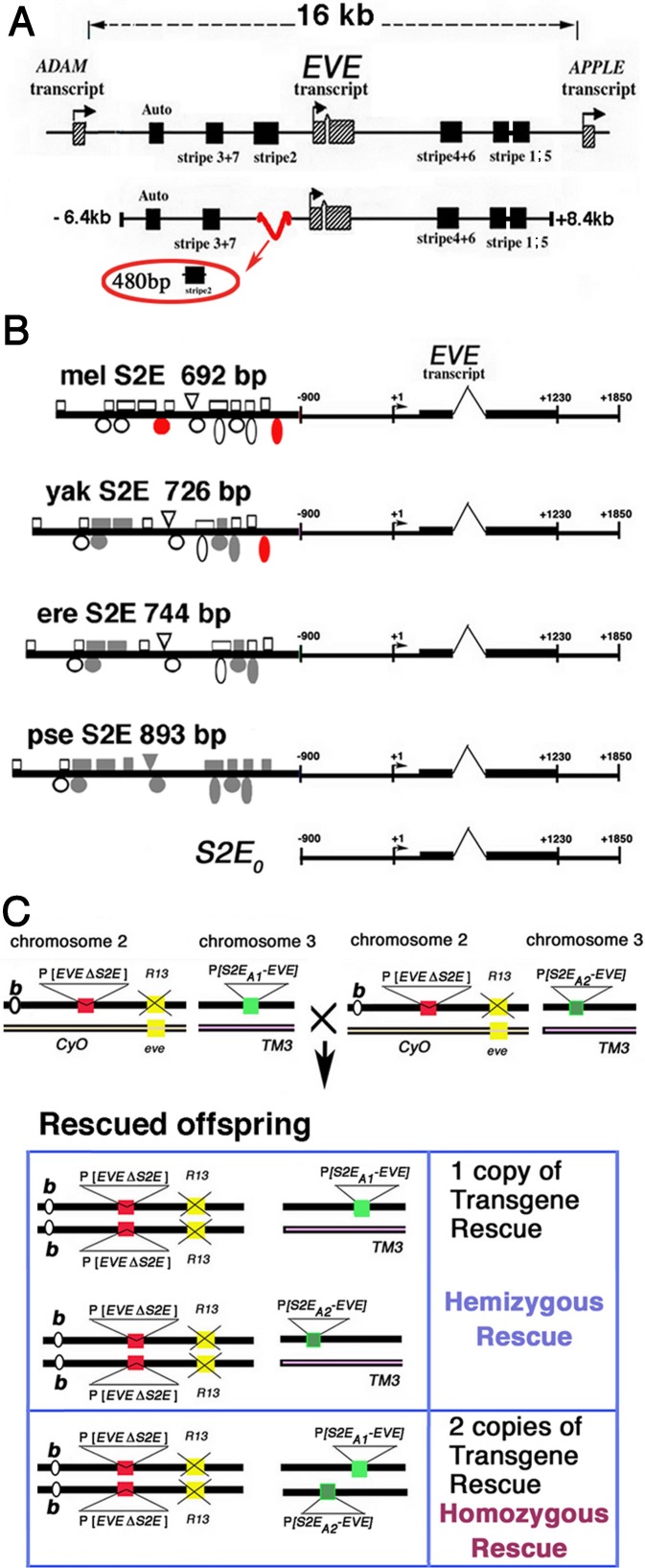
Genetic Constructs and Rescue Scheme (A) Summary map of the *eve* locus and *eve* S2E deletion transgene *(EVEΔS2E). Adam* and *Apple* are adjacent open reading frames [40]. The late element (Auto) and early stripe enhancers are shown. (B) *S2E-EVE* transgenes used to rescue *eve* function. The rescue EVE locus used is the *D. melanogaster eve* flanked by 0.9 kb of 5′ and approximately 0.6 kb of 3′ of endogenous sequence. The *S2E*_o_ *-EVE* does not have any S2E sequences and is a negative control. The known *trans*-factor-binding sites in the S2E from D. melanogaster: five bicoid (circles), three hunchback (ovals), six Kruppel (squares), three giant (rectangles), and one sloppy-paired (triangle) binding site. Symbols representing sites 100% conserved compared to D. melanogaster are open, while those diverged are shaded gray. Note the evolutionary gain of novel but functionally necessary [6] activator (bicoid and hunchback) binding sites (red) in D. melanogaster lineage. Full sequences are shown in Figures S1 and S2. (C) Example of a cross between independent rescue lines and relevant offspring genotypes for the viability assay (see Materials and Methods for details). Genetic notation *b:* mutant *black;* yellow box: native *eve;* R13 and X'd out yellow box: *eve^R13^* lethal mutant; *P(S2EΔEVE): eve −6.4* to 8.4 kb without S2E; *P(S2E*_A1_ *-EVE)* and *P(S2E*_A2_ *-EVE)* are two independent rescue-transgene inserts with S2E from species A.

The *Drosophila* gene *even-skipped (eve)* produces seven transverse stripes along the anterior–posterior (A–P) axis of a blastoderm embryo (Figure 1). Expression of these early stripes is regulated by five distinct *cis-*elements (Figure 2A). The best studied of them, the stripe 2 enhancer (S2E), contains multiple binding sites for five TFs, the activators bicoid and hunchback, and the repressors giant, Kruppel, and sloppy-paired [6,7,8]. Maternal deposition of bicoid mRNA in the anterior pole of the egg regulates expression of the other gap genes, which are expressed in broad A–P diffusion gradients. Spatiotemporal control of *eve* stripe 2 expression is brought about through the integration of these graded signals by the S2E.

We previously used a reporter transgene assay to investigate *eve* S2E functional evolution in three *Drosophila* species in addition to *D. melanogaster.* The sister taxa D. yakuba and D. erecta [9] are separated by approximately 5 million years ago (MYA), while the ancestor they share with D. melanogaster existed approximately 10–12 MYA. In contrast, D. pseudoobscura is a member of a different group and is believed to have split from the *melanogaster* clade approximately 40—60 MYA. As expected for a trait as ontogenetically important as primary pair-rule stripe formation, the temporal progression of *eve* stripe expression is nearly identical among the species (see Figure 1A–1D). This functional conservation of gene expression, however, is not reflected in patterns of sequence conservation (see Figures 2B, S1, and S2). Instead, S2E sequences from these species are substantially diverged, including large insertions and deletions in the spacers between known factor-binding sites, single nucleotide substitutions in binding sites, and even gains or losses of binding sites for the activators bicoid and hunchback.

Yet despite these evolved differences, reporter transgene analysis showed that spatiotemporal patterns of gene expression driven by S2Es of all four species are indistinguishable when placed in D. melanogaster [10], indicating that evolved changes in the enhancer have had little or undetectable impact on spatiotemporal control of gene expression. But further experiments with native and chimeric S2Es of D. melanogaster and D. pseudoobscura showed that this functional conservation required coevolved changes in the 5′ and 3′ halves of the enhancer [11], suggesting compensatory (i.e., adaptive) evolution. This functional evidence for adaptive substitution, together with indications that levels of gene expression might also differ among the four species' S2Es, raises questions about whether these orthologous enhancers are indeed functionally identical. To overcome limitations inherent in functionally interpreting the overlap of a reporter and native gene expression, here we report results of an in vivo complementation assay to investigate S2E performance. This approach allows us to put the functional equivalency hypothesis to a rigorous test.

## Results

### Strategy and Proof of Principle

First, we created a fly line, *EVEΔS2E,* in which the native *eve* S2E was deleted (see Figure 2A). We then attempted to complement, that is, rescue this lethal mutation with the introduction of a transgene, denoted *S2E-EVE,* containing an *eve* S2E from one of the four species *(D. melanogaster, D. yakuba, D. erecta,* or *D. pseudoobscura)* linked to a functional *eve* promoter and coding region (Figure 2B). This allowed us to compare both viabilities and developmental consequences among lines differing only in the evolutionary source of their S2E. By genetically manipulating rescue-transgene copy number (Figure 2C), effects of EVE abundance on viability and development could also be investigated.

**Figure 3:**
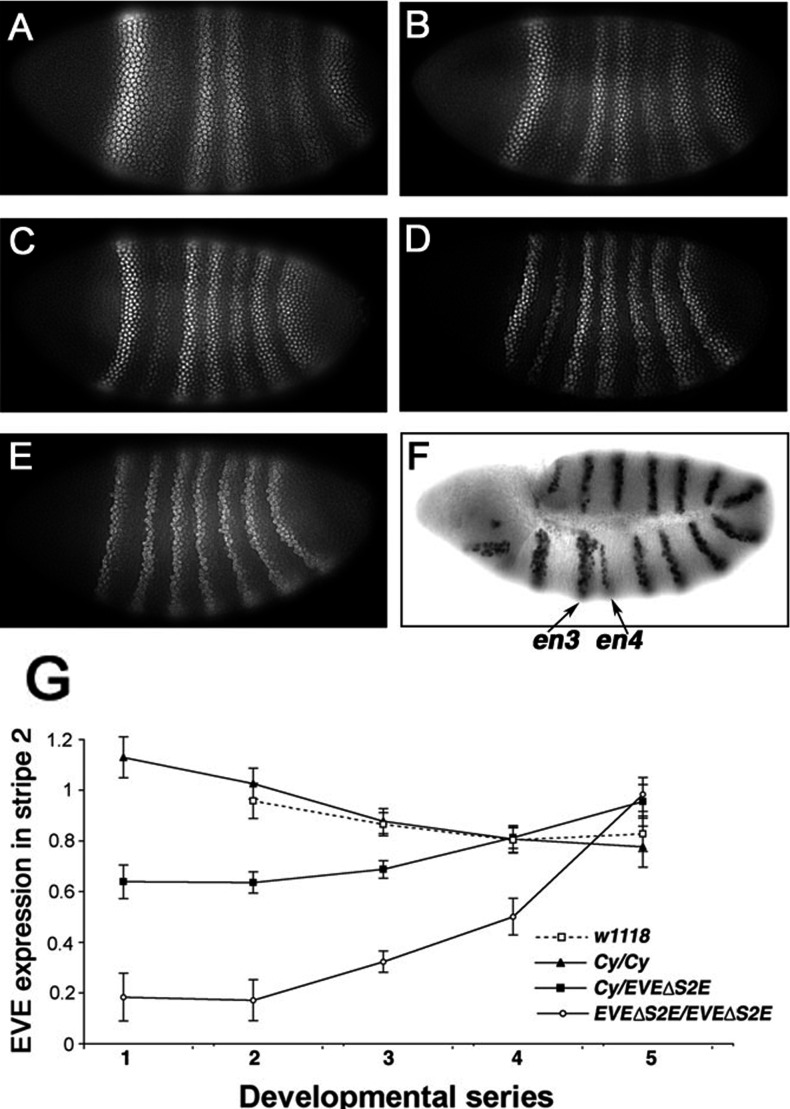
Developmental Series of EVE Abundance (A–E) Immunofluorescence labeling of time-staged early *EVEΔS2E* homozygous embryos. This developmental sequence, which corresponds roughly from the initialization of cellularization (A) to its completion (E), takes approximately 45 min at 25 ^o^C in wild-type flies [41]. (F) Expression of *en* in same genotype at stage 10. Arrows mark third and fourth *en* stripes. Note the short interval between *en* stripes 3 and 4 (parasegment 3) and the reduced fourth stripe. (G) EVE expression in stripe 2 during the developmental series around cellularization, where times 1–5 correspond to pictures in A–E. Stage 1 is early cellularization, while the process has been completed for embryos in class 5. The series is comparable to time classes 4–8 on the FlyEx Web site (http://flyex.ams.sunysb.edu/flyex/) [34]. Estimated least square means (± SE) for *EVEΔS2E/Cy* stock and wild-type line *w1118;* note the *Cy/Cy* homozygote is essentially wild-type. Early *eve* pair-rule expression is not known to be autoregulated (as occurs in postcellularization stages), and we observe a 2-fold difference in early stripe expression, with an additive component *(a)* of 0.62 and negligible dominance deviation *(d/a)* = 0.01, for the first two stages. This dosage dependency is lost after the cellularization stage (3), presumably because all embryos carry two copies of the autoregulatory element.

We created the *eve* S2E deficiency mutant by removing a 480-bp fragment corresponding to the minimal stripe 2 element (MSE; see Figure S1) from a 15-kb cloned copy of the *eve* locus [12]. A transgene containing the complete fragment is capable of rescuing *eve* null mutant flies to fertile adulthood [12]. *EVEΔS2E* is functionally a null allele for stripe 2, as evidenced by the expression of the segment polarity gene, *engrailed (en).* Establishment of *en* 14-stripe pattern is a complex process that includes involvement by *eve* early stripes [13,14]. *Eve* stripe 2 corresponds to parasegment 3, which is bordered by *en* stripes 3 and 4. We hypothesized that these *en* stripes might be developmental indicators of early *eve* stripe 2 expression. Indeed *EVEΔS2E* embryos lacking a functional S2E (Figure 3A–3F) produce a short parasegment 3 and vestigial *en* stripe 4 (Figure 3F). This defect alone is almost certainly a lethal condition.

Transgenes containing precisely orthologous S2Es from each of the four species linked to the *D. melanogaster eve* promoter and coding region were introduced onto the third chromosome. The fragment we chose to investigate is 692 bp in length in D. melanogaster (see Figure S1). It contains the central MSE, and every other previously identified TF-binding site in the S2E region. Notably, this fragment contains completely conserved sequences at its 5′ and 3′ ends in all four species, thus ensuring that we could compare precisely orthologous fragments. As expected, all four *S2E-EVE* transgenes express a single early *eve* stripe in the expected spatial location (see Figure 1E–1H).

**Figure 4:**
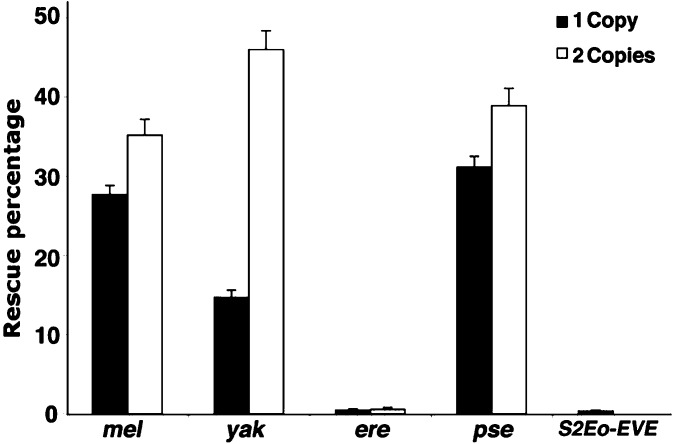
Rescue to Adulthood of *eve* Null Mutants Rescue percentages to adulthood of *EVEΔS2E* homozygotes with one or two copies of rescue construct from the four species, and the negative control, denoted on x-axis. Each bar represents percentages summarized over sexes and reciprocal crosses (full data in Table S1).

Having created the *EVEΔS2E* chromosome line and the *S2E-EVE* rescue third chromosome lines, we could then produce flies carrying *EVEΔS2E; S2E-EVE* in a doubly balanced configuration (see Figure 2C). Crossing this line with itself or with another line carrying an independent copy of the same S2E allowed us to estimate relative survival to adulthood of offspring carrying one or two copies of the rescue transgene. *EVEΔS2E* homozygotes are embryonic lethal, whereas flies carrying two copies of the *D. melanogaster S2E_mel_-EVE* transgene in an *EVEΔS2E* genetic background rescue approximately 34% of flies to adulthood (Figure 4). This is approximately the same rescue percentages found for the same genotype (*P[EVEG84],* R13), which contains the wild-type *eve* locus (including the native S2E) [12]. This implies that the fragment we used to drive stripe 2 *eve* expression is complete and that it can function normally when removed from its native context. Importantly, our negative control, *S2E_0_-EVE,* does not rescue, indicating that the rescue transgene requires this enhancer to drive *eve* stripe 2 expression.

### Functional Equivalence of the D. melanogaster and D. pseudoobscura S2Es

We evaluated the ability of *S2E-EVE* rescue constructs to complement the embryonic lethal *EVEΔS2E* deletion by estimating survival to adulthood, based on a genetic design used extensively in *Drosophila* evolutionary genetics [15]. Viability measurements were made by crossing two independent lines of each rescue transgene to reduce potential recessive fitness effects caused by the site of rescue-transgene insertion. Offspring with two copies of the transgene are doubly hemizygous; few deleterious effects of transgene insertion were observed in these flies (compare, for example, *EVEΔS2E, R13/CyO; S2E-EVE/S2E-EVE* versus *EVEΔS2E, R13/CyO; S2E-EVE/TM3* survivors in Table S1). Rescue abilities of S2Es from different species can be compared quantitatively because the viability of each *S2E-EVE* transgene is calculated relative to a standard genotype present in every cross.

S2Es from the four species exhibited large differences in rescue abilities that follow neither a phylogenetic trend nor net sequence divergence (Figure 4). The S2E of the most distantly related species, *D. pseudoobscura,* is completely conserved at only three of 18 TF-binding sites identified in D. melanogaster and is missing two of them entirely (see Figures 2B and S2). It is also nearly 25% longer due to insertions and deletions in the spacers between binding sites. Yet in terms of rescue ability it is indistinguishable from the D. melanogaster S2E.

### Functional Divergence of S2Es from Closely Related Species

Given the complete functional conservation of the D. pseudoobscura S2E, we were surprised to discover the failure of the D. erecta transgene to restore viability in *EVEΔS2E* homozygotes (see Figure 4). The inability of the doubly hemizygous *S2E_ere_-EVE* genotype to rescue cannot be due to deleterious effects of transgene insertion, because the presence of each single transgene has minimal impact on viability (see Table S1). Two additional independent transformants were also investigated, neither of which produced viable adult flies. We conclude, therefore, that the D. erecta sequence, although precisely orthologous to the D. melanogaster and D. pseudoobscura S2E fragments, is nonfunctional when placed in a D. melanogaster embryonic context.

The *D. yakuba's* S2E also exhibits a rescue defect in that two copies of the rescue transgene are required for robust rescue. Flies carrying a single copy of the D. yakuba rescue transgene are less than half as viable as flies carrying one copy of either the D. melanogaster or D. pseudoobscura rescue transgene. A smaller dosage effect on viability of approximately 20% is seen with the S2Es of D. melanogaster and *D. pseudoobscura.* Since the spatiotemporal expression of *eve* stripe 2 must be the same for flies carrying one or two copies of a transgene, *eve* stripe 2 expression level alone must have a measurable influence on fitness.

**Figure 5:**
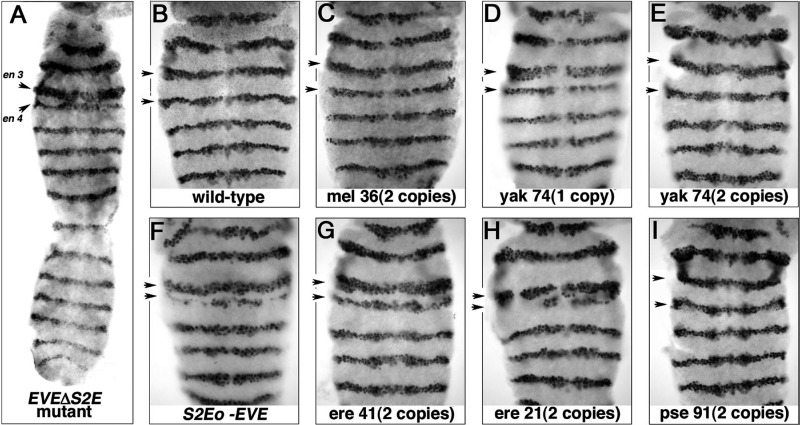
Effects on *en* Expression (A, C–I) The *en* pattern in homozygous *EVEΔS2E* and (B) wild-type *(w1118)* specimens at stages 9–11. All strains (except [B]) are homozygous for *Df(eve) P(EVEΔS2E)* second chromosomes, with the third chromosome differing only by rescue transgenes: (A) no rescue transgenes; (C) *P(mel 36)/P(mel 36)* is a *S2E_mel_-EVE* stock; (D) *P(yak 74)/TM3 Sb* and (E) *P(yak 74)/P(yak 74)* are *S2E*_yak_ *-EVE* stocks; (F) *P(S2E_o_-EVE)/P(S2E_o_-EVE)* has no S2E; both (G) *P(ere 41)/P(ere 41)* and (H) *P(ere 21)/P(ere 21)* are *S2E_ere_-EVE* transgenic stocks; and (I) *P(pse 91)/P(pse 91)* is a *S2E_pse_-EVE* stock. Note the variation in distance between third and forth *en* stripes (arrows) and relative level of *en* expression in the fourth stripe. Only the first seven parasegments of the *en* pattern are show (except in [A]). The *en* protein was visualized by an immunoperoxidase DAB reaction enhanced by nickel. *mel: D. melanogaster; yak: D. yakuba; ere: D. erecta; pse: D. pseudoobscura. S2E_o_-EVE* lacks a S2E*.*

As expected, embryos carrying one or two copies of either the D. melanogaster or the functionally equivalent D. pseudoobscura S2E rescue transgene exhibit a wild-type *en* staining pattern, indicating a normal parasegment 3 (Figure 5A–5I). In contrast, the D. erecta S2E exhibits an *en* pattern defect similar to the one produced in embryos lacking *eve* stripe 2 expression (i.e., EVEΔS2E homozygote). The inability to drive normal *en* expression provides further evidence that the D. erecta S2E is a weak (or nonfunctional) enhancer in the D. melanogaster genetic background.

The D. yakuba S2E also exhibits an *en* phenotype that correlates with its ability to rescue (Figure 5D and 5E). With two copies of the enhancer present, embryos exhibit a robust *en* stripe 4, indistinguishable from wild-type. But with only one copy present, *en* stripe 4 expression is shifted anteriorly relative to its neighbors, an indication that parasegment 3 is not forming properly. Some of these embryos survive to adulthood since we do observe one-copy adults in our viability experiment, albeit at a lower than expected percentage. Although adult flies are superficially “normal,” we can observe subtle morphological defects (mouthparts and thoracic structures) in the segments corresponding to parasegment 3.

### Differences in *eve* S2E Expression Levels

To test whether differential gene expression might be the critical functional difference between the S2Es, we quantified *eve* stripe 2 protein in early embryos. The experimental design allowed us to normalize *eve* stripe 2 expression in individually stained embryos relative to stripe 3, thus facilitating comparison across embryos and genotypes. We also developed a PCR method to ascertain the genotype of individually stained embryos.

We validated the quantification procedure by comparing *eve* stripe 2 expression levels in embryos carrying zero, one, or two copies of the S2E in its native position in a wild-type *eve* locus—that is, *EVEΔS2E/EVEΔS2E, EVEΔS2E/Cy,* and *Cy/Cy* embryos, respectively, and a homozygous *w1118* line (see Figure 3G). The expected dose dependence is observed in response to *EVEΔS2E* copy number prior to cellularization, followed by a shift to dose independence as control of *eve* stripe expression is transferred to the late (autoregulatory) element. Unexpectedly, a weak early stripe 2 (estimated to be approximately 20% of the wild-type level) can be detected in *EVEΔS2E* homozygotes; we do not know what drives this stripe.

**Figure 6:**
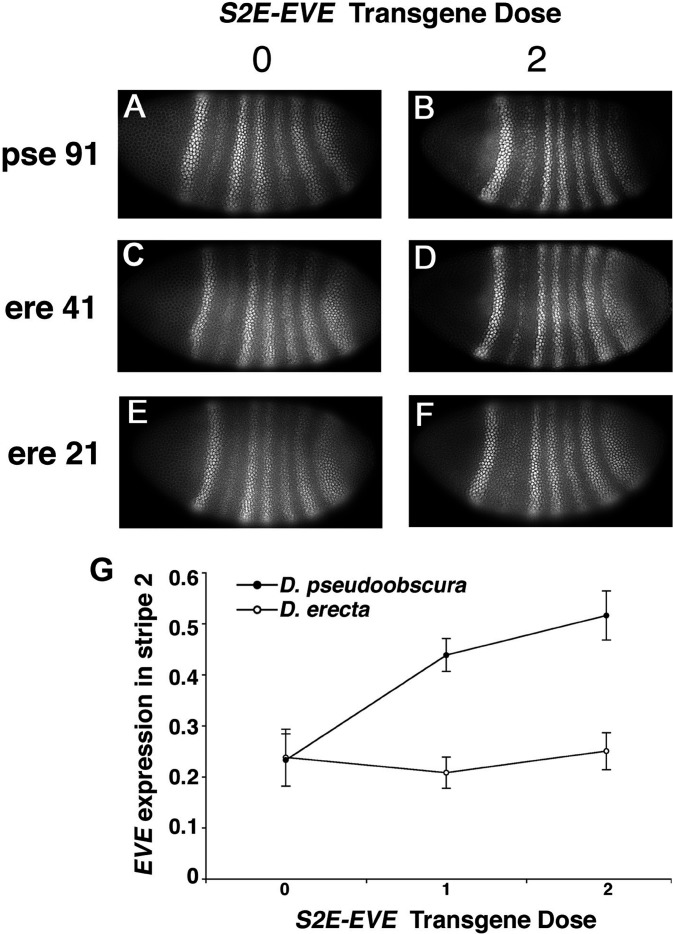
Diverged S2Es Contribute Differentially to EVE Abundance Fluorescence-labeled antibody staining of EVE in embryos with zero (A, C, and E) or two (B, D, and F) copies of rescue transgene. A dose effect is seen in D. pseudoobscura line 91, (A and B), while none is observed in D. erecta line 41 (C and D) or 21 (E and F). (G) These effects are significant when comparing EVE protein quantity (least square means ± SE) in stripe 2 (Dose × Species, *F* = 4.69_(2, 100.44)_, *p* = 0.01; see Tables 1 and S2) D. pseudoobscura (black circles, *n* = 59) and D. erecta embryos (open circles, *n* = 71). For D. pseudoobscura the estimated additive component *(a)* = 0.37 and dominance deviation*(d/a) =* 0.17.

Normalized stripe 2 expression in early embryos carrying S2Es from D. erecta and D. pseudoobscura is consistent with adult viability (Figure 6). The D. erecta S2E-driven *eve* expression is too weak to observe statistically significant expression comparing embryos containing zero, one, or two copies of the rescue transgene. Note, however, that this transgene does drive weak *eve* stripe 2 expression in a fully *eve* null background (see Figure 1G). Formally, we observe statistically significant effects of gene “dose,” S2E “species” of origin, and most notably a “dose × species” interaction on stripe 2 expression by a mixed-model analysis of variance (ANOVA) (see Tables 1 and S2). Therefore, the major functional evolutionary difference between these enhancers is likely to reside in their activation strengths.

**Table 1:**
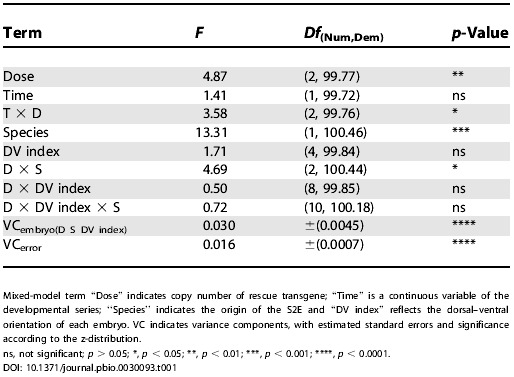
ANOVA on EVE Abundance in Stripe 2 from D. erecta and D. pseudoobscura S2E Rescue Constructs

Mixed-model term “Dose” indicates copy number of rescue transgene; “Time” is a continuous variable of the developmental series; “Species” indicates the origin of the S2E and “DV index” reflects the dorsal–ventral orientation of each embryo. VC indicates variance components, with estimated standard errors and significance according to the z-distribution

ns, not significant; *p* > 0.05; *, *p <* 0.05; **, *p <* 0.01; ***, *p <* 0.001; ****, *p* < 0.0001

## Discussion

### Evolution of Enhancer Structure–Function

The D. melanogaster S2E rescue transgene, and its considerably diverged D. pseudoobscura ortholog, each restore complete *eve* stripe 2 biological activity when placed in a genetic background lacking a native S2E. The DNA fragment we investigated, therefore, entails both the biological and evolutionary units of enhancer function. We chose this fragment based on its extensive prior characterization, including genetic, reverse genetic, and footprinting analyses [6,16,17,18]. In particular, Stanojevic et al.'s [18] TF footprinting data appear to have nicely delineated the functional enhancer.

Our previous experiments with S2Es of these two species demonstrated that both intact enhancers, but not the chimeras between them, drive the correct spatiotemporal pattern of reporter gene expression [11]. The rescue experiments reported here extend this finding by showing that the two orthologs are in fact biologically indistinguishable. These new results reinforce our contention that the phenotypic character—early stripe 2 expression—must be under stabilizing selection. The character itself remains unchanged over evolutionary time despite substitutions in nearly all the TF-binding sites, the gain and loss of some of them, and considerable change in the spacing between sites. This suggests to us that unlike proteins, where functional conservation usually means selective constraint on important amino acids (such as the active site of an enzyme), enhancers have a more flexible architecture that allows modification, and perhaps even turnover, of their “active” sites. Dissimilarities in the structure–function of enhancers and proteins result in different emergent “rules” of molecular evolution.

But the fact that the D. melanogaster and D. pseudoobscura S2Es are biologically indistinguishable does not necessarily imply that enhancer function has been evolutionary static. Rather, the similar biological activities appear to be the result of convergence. In particular, phylogenetic analysis of S2E sequences indicates that the bcd-3 binding site in D. melanogaster was acquired only recently in the lineage leading to D. melanogaster (see Figure 2B). (There are also lineage-specific deletions in the spacers flanking both sides of the bcd-3 site in the D. melanogaster lineage, which shift the proximal and distal repressors giant and Kruppel binding sites, respectively, closer to this bicoid site. These length changes may have coevolved to enable or increase local repression of this novel activator site.) The bcd-3 site was shown by Small et al. [6] to be required for MSE stripe expression. It seems likely, therefore, that the ancestral S2E lacking this binding site would not properly activate stripe 2 expression in *D. melanogaster.* Perhaps the sensitivity of the enhancer (or more precisely, the fragment investigated) to activator signals has oscillated over evolutionary time, in which case the similarity between the two distantly related species' S2Es would be an example of functional convergence.

The fact that the S2E fragment from D. erecta is essentially unresponsive to the D. melanogaster morphogen-gradient environment, but the precisely orthologous segment from D. melanogaster (and D. pseudoobscura) responds properly, proves that this fragment must contain evolved differences of *functional significance* between the species. The lack of biological activity of the D. erecta transgene in D. melanogaster should perhaps come as no surprise, however: Its lower sensitivity to activation may represent the ancestral state of the enhancer. What is surprising is the rapidity with which these functional differences evolve.

Phylogenetic footprinting of distantly related species can readily identify strongly conserved motifs [19] but runs the risk of not detecting enhancers that have retained their function but have evolved structurally. To overcome this, a technique called phylogenetic shadowing—the comparison of noncoding sequences among closely related species—has recently emerged [9]. Our results show that there is no necessary relationship between enhancer phylogenetic (or sequence) relatedness and functional similarity. Closely related species cannot be assumed to be more functionally conserved than distantly related species in enhancer structure–function.

### Why Is the D. erecta S2E Transgene Not Functional in *D. melanogaster?*

D. erecta produces a native early *eve* stripe 2. Why then does the S2E fragment from this species not produce a robust early stripe when placed in *D. melanogaster?* The first possibility is that the fragment we investigated no longer contains a functional enhancer and has been replaced by an equivalent enhancer somewhere else in the *eve* locus. This possibility can easily be ruled out: The overall architecture of the *eve* locus, including all of its 5′ and 3′ enhancers, is well conserved, and there is no new cluster of the appropriate TF-binding sites that could act as a S2E. Another unlikely possibility is that the locus has been duplicated, and the fragment we investigated has become functionally inert (i.e., equivalent to a pseudogene). There is no indication of a duplicated *eve* locus in the D. erecta genome, and all features of the *eve* locus (including its S2E) are intact and do not indicate any degeneration.

**Figure 7:**
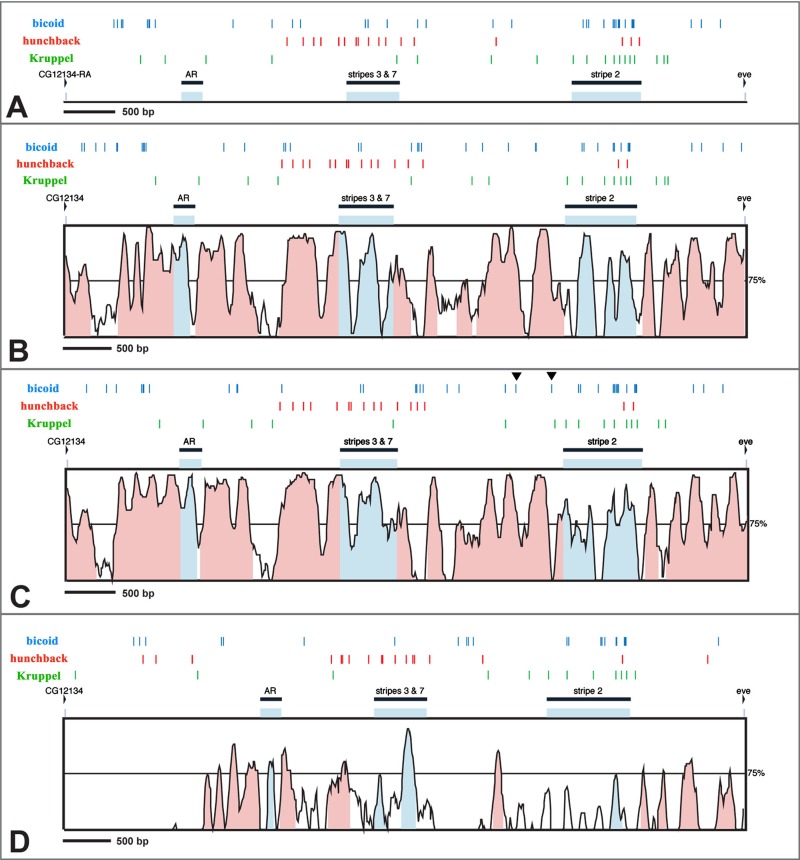
Predicted Binding-Site Composition and Sequence Conservation in the *eve* 5′ Noncoding Region (A) D. melanogaster predicted binding-site composition. (B) D. yakuba predicted binding-site composition and sequence conservation with *D. melanogaster.* (C) D. erecta predicted binding-site composition and sequence conservation with *D. melanogaster.* (D) D. pseudoobscura predicted binding-site composition and sequence conservation with *D. melanogaster.* Coordinates of functionally characterized enhancer sequences are shown in light blue, and unannotated conserved noncoding sequences are shown in pink. The coordinates of the homologous stripe 2 sequences correspond to the constructs in Figure S1, while the coordinates of the AR and stripe 3 enhancers have been estimated based on sequence conservation. Note that the scale of the genomic intervals plotted differs between panels (black bar = 500 bp). Binding sites are indicated by color; bicoid (blue), hunchback (red), and Kruppel (green).

This leads us to conclude that the D. erecta fragment used in our experiments contains the S2E. We can consider three additional possibilities. The first is that this fragment is no longer the complete biological unit, that is, novel binding sites have evolved in this species distal or proximal to this fragment, which have become assimilated into the active enhancer by a process we call accretion. As Figure 7 shows, *patser,* a binding-site prediction program [20] identifies a single potential bicoid-binding site 135 bp upstream of Block-A (Figure S1), the distal end of the D. erecta S2E transgene. This potential site contains an unconventional bicoid-binding motif, T**CAATC**CC. The next closest potential binding site is another 350 bp further upstream and also has an unconventional binding-site sequence ( A**CAATC**GG). So, although we cannot rule out the possibility that these are biologically active sites that contribute to S2E activity, they are relatively distant from the recognized S2E (and other bicoid sites), and their sequences do not have the consensus core motif ( **TAATC**). Future experiments will allow us to formally test whether this enhancer has physically expanded. If so, this would be the first documented case for accretion, the adaptive expansion of an enhancer.

The second possibility is coevolution between the D. erecta S2E and its promoter region, such that it is not capable of driving transcription properly from a D. melanogaster promoter. We also view this as unlikely for several reasons. First, prior to designing these experiments, we investigated this issue with the core promoters and S2Es of D. melanogaster and D. virilis (which is an outgroup to the species studied). We could detect no difference in spatial or temporal expression of each S2E with either promoter (Ludwig, unpublished data). Second, the core promoter regions of D. melanogaster and D. erecta are highly conserved, including complete preservation of both the TATAA and the GAGA site. Indeed there are only four nucleotide differences (and no indels) between the species in a 150-bp stretch containing these sites. Finally, one might expect most functional changes in the core promoter to be pleiotropic, given the presence of more than a dozen other separable enhancers in the *eve* locus, and therefore to be selected against.

The final possibility is that the D. erecta S2E fragment does contain the entire biological enhancer, but that the *trans*-acting environment—the morphogen gradients to which the enhancer responds—differ between the species, causing the enhancers to have evolved to accommodate the differences. In other words, the sensitivity, or set point, of the binary (on–off) switch function has coevolved with the *trans*-acting environment in order for the S2E to maintain the appropriate response to evolved activation inputs. This hypothesis implicates, in particular, evolutionary shifts in the bicoid and/or hunchback activator gradients. As noted above, there has been a lineage-specific addition of the functionally required bcd-3 binding site [6] in D. melanogaster that is not present in any of the other species. Second, there is also a lineage-specific loss of the hunchback-1 (hb-1) site in D. erecta (which may be present in its sister taxon, D. yakuba). We propose that the lack of sites for these activators, and the presence of a species-specific six-base-pair insertion in the overlapping hb-2/kr-2 binding sites (Figure S1) reduces the ability of the D. erecta enhancer to respond to the activator gradients of *D. melanogaster.*

This hypothesis predicts stronger activator gradients in D. erecta than in *D. melanogaster.* Although we have not yet investigated this possibility directly, we note that native *eve* blastoderm stripes do not reside in the same physical locations in embryos of the two species, but rather are displaced posteriorly in D. erecta compared to D. melanogaster (compare Figure 1A and 1C). A similar effect can be mimicked in D. melanogaster with the addition of extra copies of *bicoid* gene [21], which shifts the morphogen gradient posteriorly.

The possible independence of spatiotemporal and rheostat activities [22] relates to a long-standing issue in evolutionary genetics—whether developmental constraints are “tunable” [23]. One school holds that features of development are strongly canalized and that deviation from this path will be strongly selected against. An opposing school holds that these developmental constraints can always be overcome by selection. The *eve* S2E may exhibit elements of both an immutable developmental constraint and a smoothly evolving trait. Primary pair-rule stripes, such as those laid down by *eve* in developing blastoderm embryos, establish the positional landmarks that will eventually demarcate segmental identities in the fly. This complex functional network established by gap and pair-rule gene expression imposes strong constraints on spatiotemporal patterns of regulatory gene expression. The S2E must, for example, produce a stripe equidistant from stripes 1 and 3, and at a specific location with respect to other pair-rule genes.

The potential for evolutionary change in the S2E set point or output, on the other hand, may be much less constrained. Genetic variability in the gain and loss of binding sites, polymorphism in binding sites leading to variation in TF binding, and modulation of TF interactions through changes in spacing between binding sites should allow for nearly continuous fine-tuning of these functions. Enhancers should be able to adapt to change in their *trans*-acting environment. Why might the *trans*-acting environment for the S2E be evolving? Residing at the head of the hierarchal cascade driving the formation of the A–P axis, perhaps the bicoid morphogen gradient is less constrained than the expression of downstream genes that are more deeply embedded in the interaction network. Or, perhaps there has been natural selection acting on traits such as egg shape, size, or ovariole morphology, leading to changes in the bicoid-diffusion gradient. Egg number and size are, after all, fundamental evolutionary trade-offs. With a properly designed genetic experiment it should be possible to test these hypotheses.

### Enhancer Evolution in Relation to Speciation

Postmating isolation is the final step in the speciation process because it involves genetic changes between incipient species that cause hybrid inviability or infertility. Hybrid breakdown is likely to involve evolutionary changes in at least two interacting genes [24,25]. According to this model, the coevolution of a “speciation” gene with its partner(s) allows it to remain functional in its native background but lethal in the hybrid background. One of the mysteries of this process is the regularity and quickness with which molecular incompatibility arises. Thus, for example, exceedingly closely related species, such as *D. simulans, D. sechellia* and *D. mauritiana,* whose common ancestors occurred less than 1 MYA, nevertheless, have evolved postmating isolation involving perhaps hundreds of genes [26]. The question is what components of the genome are involved in this coevolutionary conspiracy?

*Cis-*regulatory modules and the *trans*-acting TFs with which they interact provide abundant genetic substrates for coevolution leading to hybrid sterility and inviability [2,5]. If our experiments have captured the entire biological S2Es of D. erecta and *D. yakuba,* then the results suggest that this coevolution could involve changes in expression patterns or levels, rather than changes in the protein sequences of the *trans*-acting factors. The attractiveness of this hypothesis lies in the fact that there must be many more *cis-*regulatory modules in a eukaryotic genome than there are encoded proteins. Thus there is ample opportunity for the coevolution of enhancers and *trans*-factors to produce lethal interactions in hybrids, which may explain the abundance of lethal interactions between closely related species.

The regulation of development is often modeled as a logic circuit, with *cis-*regulatory sequences functioning as switches controlling information flow. The long-term functional preservation of both the spatiotemporal and the activation strengths of the D. melanogaster and D. pseudoobscura S2Es speaks to the general conservation of this genetic network in fruit fly development. Our results also provide an indication that the stoichiometry of the regulatory components could matter critically for normal development, at odds with theoretical predictions [27]. Epistatic changes accompanying interspecific inviability and sterility may therefore arise more readily as a consequence of quantitative shifts in gene expression than as a result of alterations in the topology of the developmental circuits.

## Materials and Methods

### 

#### *Drosophila* strains

*Df(2R)eve*: (Df[eve]) and *eve^R13^* (R13): Df[eve] is a deficiency that includes at least three lethal complementation groups [28]. The R13 is null mutation that truncates the protein within the homeodomain [12].

These lethal mutations were balanced over marked balancer chromosome *CyO P(hb-lacZ)* to allow identification of mutant embryos by immunostaining for β-galactosidase or by PCR analysis for β-galactosidase gene.

#### Analysis of embryos

Histochemical staining with guinea pig polyclonal a-Eve [29] at 1:1,000 dilution, or with a-En monoclonal 4D9 [30] at 1:10 dilution was visualized using HRP-DAB enhanced by nickel [31].

Fluorescent staining of *Drosophila* embryos with polyclonal a-Eve [29] at 1:1,000 dilution was visualized using Alexa Fluor-594 goat antiguinea pig IgG antibodies (Molecular Probes, Eugene, Oregon, United States) at 1:400 dilution.

Optical Z-sectioning (0.8-μm/step) of fluorescent embryos was carried out using motorized microscope Axioplan2 (Carl Zeiss, Thornwood, New York, United States), Hamamatsu C4742–95 camera (Hamamatsu City, Japan), and “Openlab” software (Improvision, Emeryville, California, United States).

#### Genotyping

Individual embryos were genotyped after imaging, using a PCR method (three pairs of fluor-labeled PCR primers) and Beckman Coulter CEQ^™^ 8000 genetic analysis system for PCR fragment analysis (Allendale, New Jersey, United States; for detailed protocol see Supporting Information). Three sets of fluor-labeled primers (Proligo, Boulder, Colorado, United States) were used for PCR: *P(hb-lacZ):* (1) marker for SM1(CyO) balancer second chromosome (156 bp), +106 adh: 5′ TCTGGGAGGCATTGGTCTGGA 3′ and −241 lac: 5′ CGGGCCTCTTCGCTATTACG3′, (2) *EVEΔS2E* and native *eve* locus: 592 bp and 113 bp markers for native S2E and S2E with 480 bp MSE deleted, respectively, +23 Df: 5′ TAACTGGCAGGAGCGAGGTATC3′ and −115 Df: 5′ CTCGCGGATCAGGGCTAAGT3′, (3) DMPROSPER, 3rd chromosome microsatellite marker for *TM3 Sb* balancer and rescue transgene-containing chromosome III, DMPROSRER F: 5′ CGGTACAAAGTGTGTGTTC3′ and DMPROSRER R: 5′ GACTTTTAAACATTTAAGATTAATTCC3′.

#### S2E mutant construct *(EVEΔS2E)*

A pCaSpeR-based vector containing wild-type *eve* genomic DNA from −6.4 to +8.4 kb (EVEG84) was provided by Miki Fujioka [12]. The deficiency *eve* S2E *(EVEΔS2E)* mutation was created by deleting the region from −1554 (BstEII) to −1073 kb (BssHII) relative to *eve* transcription start site (see Figure 2).

#### *S2E* rescue constructs *(S2E-EVE)*

The S2Es used in this study were cloned previously [10], and their accession numbers are given as supplemental information. The sequences employed in this study are presented in Figure S1; they all begin and end at conserved sequences flanking the S2E (blocks A and B).

The *S2E*-*eve* rescue transgenes based on our modification of the E-eve pCaSpeR vector provided by M. Fujioka [32] were constructed as follows: 2.76-kb *eve* CaSpeR (negative control construct, *S2E_0_-EVE*) carries the D. melanogaster wild-type *eve* genomic DNA fragment from −913 (FspI) to +1.85(MluI) relative to transcription start site, which includes 913 bp intact *eve* 5′ upstream region, protein-coding sequence, and the polyadenylation site. A unique restriction site PmeI was created instead of the FspI site, so that S2Es from different species could be cloned into the 2.76-kb *eve* CaSpeR vector by using unique PmeI and NotI sites in the proper orientations. The entire *eve* region in rescue constructs from D. melanogaster and *D. erecta,* including both the S2E and *eve* genomic DNA fragment from −913 to +1.85, were confirmed by sequencing.

#### P-transformation

P-element-mediated germline transformation was carried out as described by Rubin and Spradling [33]. A homozygous viable stock with the S2E deficiency transgene P*(EVEΔS2E)* on chromosome II was recombined onto *eve* mutant chromosomes—either *Df(eve)* or eve^R13^—to create lines that can only drive *eve* expression from the *EVEΔS2E* transgene. These chromosomes are maintained as balanced stocks.

Rescue-transgene lines *(S2E-EVE)* were chosen for use in the study if they were homozygous viable and were on chromosome III. Between 2 and 4 independent stable transformed lines were generated for each rescue construct and were examined for rescue ability to adulthood and for local *eve* and *en* pattern rescue.

#### Rescue of *eve* function to adulthood

Each transgenic rescue line was crossed into the *eve^R13^* (R13) P*(EVEΔS2E)* mutant background, generating flies of the genotype *w; b* R13 P*(EVEΔS2E)/CyO;* P*(S2E_A1_-EVE)/TM3 Sb.* These flies were reciprocally crossed with flies of the genotype *w; b* R13 P*(EVEΔS2E)/CyO;* P*(S2E_A2_-EVE)/TM3 Sb.* A1 and A2 indicate independent transformed lines with the identical rescue construct (see Figure 2). Adults were scored for both a wild-type wing phenotype (non-CyO) and the black *(b)* phenotype (indicating R13 homozygotes).

For each reciprocal cross 50 healthy virgin females from one strain were mated in a standard culture bottle with 100 healthy males from the other. Parents were transferred to fresh culture bottles every 3 d for 24 d. The emerging adult offspring were collected every day from the culture bottles for a period of 10 d for scoring. This approach ensured that mutants with slow development rates were counted. The cultures were kept at 25 °C.

Viability of flies carrying one or two copies of the rescue transgene was measured relative to the number expected based on the count of flies carrying one copy of the dominantly marked second and/or third chromosome. This genetic design allowed us to estimate viability effects of transgene insertion independent of transgene rescue ability by comparing genotypes carrying one (hemizygous) or two (doubly hemizygous) copies of the rescue transgene in genotypes carrying a wild-type *eve* locus (i.e., *EVEΔS2E, R13/CyO; S2E-EVE/TM3* versus *EVEΔS2E, R13/CyO; S2E-EVE/S2E-EVE;* see Table S1). Viabilities of rescue genotypes were calculated by comparing the number of adult survivors in *EVEΔS2E* homozygotes *(EVEΔS2E, R13/EVEΔS2E,R13)* relative to the number of survivors in the corresponding genotype with one copy of a functional *eve* locus *(EVEΔS2E, R13/CyO; S2E-EVE/S2E-EVE).*

#### Localized rescue of *eve* and *en* expression patterns

Each transgenic rescue line was crossed into the *Df(eve)* P(*EVEΔS2E*) mutant background, generating stock *w; Df(eve)* P*(EVEΔS2E)/CyO* P*(hb-lacZ)*; P*(S2E-EVE)/TM3 Sb.*

The embryos of these stocks were stained with anti-Eve and anti-Engrailed antibodies to determine the pattern and level of genes expression. The embryos inspected for *en* were dissected flat, and the proctodeum and posterior midgut removed, anterior up, and viewed from the ventral side (see Figure 3F for undissected specimen). Genotypes of the embryos were determined after images were taken, as described above.

#### Image processing and EVE quantification

*eve* stripes 1, 3, 4, 5, 6, and 7 are always produced from the *EVEΔS2E* locus in our experimental flies, whereas *eve* stripe 2 comes primarily from the independent *S2E-EVE* rescue transgene. This allowed us to compare stripe 2 expression levels in different embryos and genotypes by measuring stripe 2 expression relative to an adjacent stripe. We chose the adjacent stripe 3 as a reference, as it has similar temporal and quantitative expression, and we report the stripe 2 expression relative to stripe 3. Image stacks (0.8 μm) were deconvoluted in Huygens Essential (version 2.5.0 from Scientific Volume Imaging, Hilversum, the Netherlands) and 3–5 images in the focal plane collapsed to a single image in Image J (version 1.30, free at http://rsb.info.nih.gov/ij/). All were subjected to the same background subtraction and a section corresponding to stripes 1 to 4 in the middle section of the embryo cropped out and saved as a raw pixel file for analysis in Mathematica 5 (www.wolfram.com). The location of each stripe was estimated by fitting second order curves to local maxima/minima of smoothed data, identifying stripes 1 through 4 and the interleaving troughs. The approach worked reasonably, as of 205 embryos surveyed in the rescue experiment only 30 were rejected because the algorithm could not detect a stripe 2. The reason for rejection was in the majority of cases not the lack of stripe 2 expression in individuals homozygous for the *EVEΔS2E,* but rather the absence of a detectable trough between stripes 1 and 2 during early stages of stripe formation.

The fitted curves were superimposed on the raw data and used as guides for the summing of the signal intensity, from the middle 25%, 50%, or 75% of the stripes and the 25% of the troughs. These percentages were derived from the total length between stripes 1 and 4, and therefore represent comparable geometric areas. This is important as we proceed to compare abundances in stripes 2 and 3. Percentages of stripes were used to account for size variation as variables extracted on the basis of absolute pixels were noisier. The measurements were summed from 5 to 11 sections per embryo, each 30-pixels high. The dependent variables analyzed were ratios of the measured fluorescence in stripe 2 (P2) over stripe 3 (P3) after adjusting the measured background in the separating trough (T2), in general form: Y = (P2 − T2)/(P3 − T2). These variables were derived for the middle 25%, 50%, and 75% of the stripes, but all yielded similar results, and we report on the 75% case. For every embryo we documented a DV index, indicating its degree of rotation along the dorsal–ventral axis (divided into five categories, dorsal, dorsal–middle, middle, middle–ventral, and ventral). In addition we placed the embryos into a series (five classes) around cellularization corresponding to approximately 45 min of development, when the EVE stripes originate and take form (see Figure 3A–3E). The series is based on classes 4–8 for the 14th cleavage cycle available on the FlyEx Web site at http://flyex.ams.sunysb.edu/flyex/ [34].

#### ANOVA on EVE expression

Mixed-model ANOVA was fitted in SAS v8.2 (2002; SAS Institute, Cary, North Carolina, United States) with the main effects of “dose,” that is, how many copies of rescue construct; “species,” designating the origin of the S2E; and a “DV index,” which accounts for orientation, along the dorsal–ventral axis, of measured embryos. Also, “time,” indicating the inferred stage of development, was used as a covariate. Finally, as each embryo was measured several times, we avoided pseudoreplication by nesting the random variable “embryo” within the fixed effects.


Y = μ + Dose + Species + Time + DV index + D × S + D × T + D × DVI + D × S × DVI + Embryo (D × S × DVI) + ɛ

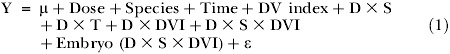



The results were identical if the multiple recordings on each embryo were designated as repeated measures within the Proc Mixed statement. We also applied a generalized linear model to the least square mean of the phenotypic values for each embryo. Again results were in accord (see Tables 1 and S2). A related model, excluding “species” terms, was used to evaluate the “dose” effects in the *EVEΔS2E* stock. We also investigated the general capacity of the D. pseudoobscura construct to reconstitute the activity of the endogenous gene. The stock with *EVEΔS2E* carried over a *Cy* balancer was used, and embryos of all three genotypes were collected. The mixed model was constructed in the same way, adding “experiment” as a term. All stocks were constructed to have the same genetic background, and we predicted that individuals homozygous for the *EVEΔS2E* in both datasets would have similar expression. This was corroborated by ANOVA with a reduced mixed model, F_(1, 52.54)_ = 1.70, *p* = 0.20, *n* = 71. Similarly, the EVE expression of the homozygous balancer could not be distinguished from a wild-type strain *w1118:* F_(1, 63.71)_ = 1.00, *p* = 0.32, *n* = 76 (see Figure 3G). Using standard quantitative genetic theory [35], we assessed the genotypic effects of the S2E on *eve* expression in the D. pseudoobscura transgenes and the endogenous gene. The small sample size prohibited formal tests of deviations from additivity.

#### Sequence analysis

Comparative sequence analysis was conducted with the Avid-Vista suite (http://www-gsd.lbl.gov/vista/, using default parameters [36,37]. Binding-site prediction was performed using *patser* [20] with identical command-line arguments and position-weight matrices used by Berman et al. [38]. Cutoffs for display of predicted binding sites were set to recover all known binding sites in the D. melanogaster S2E–ln(P) = −6.1 for *bicoid,* ln(P) = −8.06 for *hunchback,* and ln(P) = −6.65 for *Kruppel.*

## Supporting Information

Figure S1 Alignment of *eve* S2E Regions from Four Species of *Drosophila*
Gaps in aligned sequences are indicated by dashes. The binding sites in D. melanogaster for the *trans*-acting factors, bicoid (bcd), hunchback (hb), Kruppel (kr), sloppy-paired (slp), and giant (gt), are shown above the sequence. The sequences from the four species begin and end at completely conserved sequences, indicated by blocks A and B, flanking the enhancer. *mel: D. melanogaster; yak: D. yakuba; ere: D. erecta; pse: D. pseudoobscura.*
(524 KB JPG).

Figure S2 Trans-Factor-Binding Sites in D. melanogaster S2E and Homologous Sequences from Three Other *Drosophila* Species Binding sites are for five proteins are designated; bicoid (bcd), hunchback (hb), Kruppel (kr), sloppy-paired (slp), and giant (gt). N/A: no homologous sequence identified. *mel: D. melanogaster; yak: D. yakuba; ere: D. erecta; pse: D. pseudoobscura.* The figure is expanded from Ludwig et al. [10] and Ludwig [2].
(954 KB JPG).

Protocol S1 Additional Materials and Methods (29 KB DOC).

Table S1 Viability to Adulthood Adult viability of individuals from reciprocal crosses between two independent transgenic lines with *eve* S2E from the four species and from a negative control *(S2E_0_-EVE).* See Figure 2 for constructs and crossing scheme. With Mendelian segregation of the 2nd and 3rd chromosomes, ratios of 4:2:2:1 for the genotypic classes are anticipated. This was used to calculate expected counts and rescue percentages (in brackets) for the classes with one and two copies of rescue constructs. Adjusted rescue percentages, which account for possible detrimental effects of two hemizygous transgenic inserts, are also reported. These data summarized over sexes are represented in Figure 4.
(31 KB DOC).

Table S2 ANOVAs on EVE Abundance in Stripe 2 ANOVAs on EVE quantity. (A) Analysis on amounts of EVE in stripe 2 D. erecta and D. pseudoobscura rescue data (B) on the rescue data for all four species and (C) the *EVEΔS2E/Cy* stock. Mixed-model ANOVA (left) fitted the multiple measures per embryo as random. Generalized linear model (right) was implemented on the least square means calculated for each embryo. “Dose” indicates the copy number of rescue transgene per embryo; “Time” indicates a continuous variable of the developmental series; “Species” indicates the origin of the S2E; “DV index” indicates orientation of each embryo along the dorsal–ventral axis. VC indicates variance components for “embryos” or residual error, with estimated standard errors and significance according to the *z*-distribution (VCs can only be estimated with a mixed model). ns, *p* > 0.05; *, *p <* 0.05; **, *p <* 0.01; ***, *p <* 0.001; ****, *p* < 0.0001.
(93 KB DOC).

### Accession Numbers

The GenBank (http://www.ncbi.nlm.nih.gov/) accession numbers for the S2Es sequences used in genetic constructs are AF042712 *(D. pseudoobscura),* AF042711 *(D. erecta),* AF042710 *(D. yakuba),* and AF042709 *(D. melanogaster).* The sequences for genomic alignments of the eve 5′ region were extracted from GenBank (D. melanogaster genomic scaffold, AE003831, and accessions AY190939 and AY190942 for D. erecta and *D. pseudoobscura,* respectively [39]). The exception was the *D. yakuba,* sequence, which came from the 7 April 7 2004 version available on the Washington University genome page (http://genome.wustl.edu/).

## Abbreviations

bcd-3: bicoid-3
*en*: *engrailed*
*eve*: *even-skipped*
hb-1: hunchback-1
MSE: minimal stripe 2 element
MYA: million years ago
S2E: stripe 2 enhancer
TF: transcription factor
