# Supplementary material for: Functional Evolution of a cis-Regulatory Module
Source: PLoS Biol. 2005 Mar 15;3(4):e93. doi: 10.1371/journal.pbio.0030093 (PMC1064851; doi:10.1371/journal.pbio.0030093)
Supplement: Supplementary file 1 [file pbio.0030093.sd001.doc]

**Protocol S1. PCR-based genotyping of individual Drosophila embryos after formaldehyde fixation, histochemical (alkaline phosphatase and brown or black HRP reaction), or fluor staining ( with using anti-fade agent like a n-Propyl gallate) , and microscopy examination.**

1. Pry open the cover glass to expose stained embryo (in 70% or 90% glycerol solution prepared using 1x PBS).
2. Wash embryo twice in 50-60μl 70% glycerol and then in 45-50μl Tris-HCl, pH 8.0: 0.05% Tween-20.
3. Transfer embryo to a PCR tube in 1μl of 10mM Tris-HCl, pH 8.0: 0.05% Tween-20 solution.
4. Add 4μl of Proteinase K solution, 0.56mg/ml in 1X PBS (Roche; Cat#: 1373196).
5. Incubate 2 hour at 50°C.
6. Add 20μl of Buffer #3 solution:

2.5μl 10x Buffer #3 (Expand Long Template PCR System; Cat # 1742663, Roche)

17.5μl water

7. Incubate 90 sec at 100°C in PCR machine; place on ice.
8. PCR:

10μl embryo sample

H_2_0 (20μl final volume)

1μl of 10x Buffer #3

1.6μl of 10mM dNTP’s (2.5mM each)

Primer cocktail (1μl of 20pMol each)

0.1 μl of Taq (5 units/μl)

9. Run for 35 cycles. The program for PCR:

Start: T=95C for 1min;

Denaturation: T=95C for 15 sec

Annealing: T=58C for 30 sec,

Extension: T=72C for 20 sec.

End: T=72C for 7min

10. Analyze PCR products by using 1.5% agarose gel electrophoresis and “Beckman Coulter CEQ^TM^8000 Genetic Analysis System “ for PCR Fragment Analysis.

Primers: Three sets of fluor-labeled primers (Proligo) were used for the PCR reaction:

1) Marker for SM1(CyO) balancer second chromosome (156 bp), -

+106adh (fluor): 5’TCTGGGAGGCATTGGTCTGGA 3’ and

-241lac: 5’CGGGCCTCTTCGCTATTACG3’,

2)	*VEΔ S2E* and native *eve* locus: 592 bp and 113 bp markers for native S2E and S2E with 480 bp MSE deleted, respectively, -

+ 23Df (fluor): 5’TAACTGGCAGGAGCGAGGTATC3’ and

-115Df: 5’CTCGCGGATCAGGGCTAAGT3’.

3) DMPROSPER, 3^rd^ chromosome microsatellite marker for TM3Sb balancer and rescue transgene-containing chromosome III, -

DMPROSRER F (fluor): 5’CGGTACAAAGTGTGTGTTC3’ and

DMPROSRER R: 5’GACTTTTAAACATTTAAGATTAATTCC3’.
