# Supplementary material for: Functional Evolution of a cis-Regulatory Module
Source: PLoS Biol. 2005 Mar 15;3(4):e93. doi: 10.1371/journal.pbio.0030093 (PMC1064851; doi:10.1371/journal.pbio.0030093)
Supplement: Supplementary file 2 [file pbio.0030093.st001.doc]

Table S1. Viability to Adulthood
