# Supplementary material for: Functional Evolution of a cis-Regulatory Module
Source: PLoS Biol. 2005 Mar 15;3(4):e93. doi: 10.1371/journal.pbio.0030093 (PMC1064851; doi:10.1371/journal.pbio.0030093)
Supplement: Supplementary file 3 [file pbio.0030093.st002.doc]

Table S2.

Table S2. ANOVAs on EVE Abundance in Stripe 2

|  |  | Mixed Model | | | Generalized Linear Model | | |
| --- | --- | --- | --- | --- | --- | --- | --- |
| A) | Term | *F* | *df*_(Num,Dem)_ | *p*-value | *F* | *df*_(Num,Dem)_ | *p*-value |
|  | Dose | 4.87 | (2, 99.77) | ** | 4.89 | (2, 99) | ** |
|  | Time | 1.41 | (1, 99.72) | ns | 1.47 | (1, 99) | ns |
|  | T X D | 3.58 | (2, 99.76) | * | 3.60 | (2, 99) | * |
|  | Species | 13.31 | (1, 100.46) | *** | 13.40 | (1, 99) | *** |
|  | DV index | 1.71 | (4, 99.84) | ns | 1.72 | (4, 99) | ns |
|  | D X S | 4.69 | (2, 100.44) | * | 4.74 | (2, 99) | * |
|  | D X DV index | 0.50 | (8, 99.85) | ns | 0.51 | (8, 99) | ns |
|  | D X DV index X S | 0.72 | (10, 100.18) | ns | 0.72 | (10, 99) | ns |
|  |  |  |  |  |  |  |  |
|  | *VC_embryo(D S DV index)_* | 0.030 | ± (0.0045) | **** | . | . | . |
|  | *VC_error_* | 0.016 | ± (0.0007) | ** | . | . | . |
|  |  |  |  |  |  |  |  |
| B) | Term | *F* | *df*_(Num,Dem)_ | *p*-value | *F* | *df*_(Num,Dem)_ | *p*-value |
|  | Dose | 5.81 | (2, 124.66) | ** | 5.84 | (2, 124) | ** |
|  | Time | 2.01 | (1, 124.47) | ns | 2.08 | (1, 124) | ns |
|  | T X D | 3.77 | (2, 124.5) | * | 3.79 | (2, 124) | * |
|  | Species | 5.30 | (3, 126.27) | ** | 5.32 | (3, 124) | ** |
|  | DV index | 1.31 | (4, 124.86) | ns | 1.31 | (4, 124) | ns |
|  | D X S | 3.24 | (6, 126.19) | ** | 3.27 | (6, 124) | ** |
|  | D X DV index | 1.03 | (8, 124.91) | ns | 1.04 | (8, 124) | ns |
|  | D X DV index X S | 0.89 | (24, 125.17) | ns | 0.90 | (24, 124) | ns |
|  |  |  |  |  |  |  |  |
|  | *VC_embryo(D S DV index)_* | 0.031 | ± (0.0042) | **** | . | . | . |
|  | *VC_error_* | 0.018 | ± (0.0007) | **** | . | . | . |
|  |  |  |  |  |  |  |  |
| C) | Term | *F* | *df*_(Num,Dem)_ | *p*-value | *F* | *df*_(Num,Dem)_ | *p*-value |
|  | Dose | 43.04 | (2, 120.51) | **** | 42.78 | (2, 121) | **** |
|  | Time | 10.40 | (1, 120.25) | ** | 10.41 | (1, 121) | ** |
|  | T X D | 25.71 | (2, 120.49) | **** | 25.56 | (2, 121) | **** |
|  | DV index | 1.09 | (4, 120.35) | ns | 1.08 | (4, 121) | ns |
|  | D X DV index | 2.54 | (8, 120.42) | * | 2.52 | (8, 121) | * |
|  |  |  |  |  |  |  |  |
|  | *VC_embryo(D DV index)_* | 0.026 | ± (0.0037) | **** | . | . | . |
|  | *VC_error_* | 0.019 | ± (0.0009) | **** | . | . | . |
